# Epithelial and Neutrophil Interactions and Coordinated Response to *Shigella* in a Human Intestinal Enteroid-Neutrophil Coculture Model

**DOI:** 10.1128/mbio.00944-22

**Published:** 2022-06-02

**Authors:** Jose M. Lemme-Dumit, Michele Doucet, Nicholas C. Zachos, Marcela F. Pasetti

**Affiliations:** a Department of Pediatrics, Center for Vaccine Development and Global Health, University of Maryland School of Medicine, Baltimore, Maryland, USA; b Department of Medicine, Division of Gastroenterology and Hepatology, Johns Hopkins University School of Medicine, Baltimore, Maryland, USA; Emory University School of Medicine

**Keywords:** enteroid, host-pathogen interactions, human, intestinal mucosa, neutrophils, *Shigella*, epithelial cells, innate immunity

## Abstract

Polymorphonuclear neutrophils (PMN) are recruited to the gastrointestinal mucosa in response to inflammation, injury, and infection. Here, we report the development and the characterization of an *ex vivo* tissue coculture model consisting of human primary intestinal enteroid monolayers and PMN, and a mechanistic interrogation of PMN-epithelial cell interaction and response to *Shigella*, a primary cause of childhood dysentery. Cellular adaptation and tissue integration, barrier function, PMN phenotypic and functional attributes, and innate immune responses were examined. PMN within the enteroid monolayers acquired a distinct activated/migratory phenotype that was influenced by direct epithelial cell contact as well as by molecular signals. Seeded on the basal side of the intestinal monolayer, PMN were intercalated within the epithelial cells and moved paracellularly toward the apical side. Cocultured PMN also increased basal secretion of interleukin 8 (IL-8). *Shigella* added to the apical surface of the monolayers evoked additional PMN phenotypic adaptations, including increased expression of cell surface markers associated with chemotaxis and cell degranulation (CD47, CD66b, and CD88). Apical *Shigella* infection triggered rapid transmigration of PMN to the luminal side, neutrophil extracellular trap (NET) formation, and bacterial phagocytosis and killing. *Shigella* infection modulated cytokine production in the coculture; apical monocyte chemoattractant protein (MCP-1), tumor necrosis factor alpha (TNF-α), and basolateral IL-8 production were downregulated, while basolateral IL-6 secretion was increased. We demonstrated, for the first time, PMN phenotypic adaptation and mobilization and coordinated epithelial cell-PMN innate response upon *Shigella* infection in the human intestinal environment. The enteroid monolayer-PMN coculture represents a technical innovation for mechanistic interrogation of gastrointestinal physiology, host-microbe interaction, innate immunity, and evaluation of preventive/therapeutic tools.

## INTRODUCTION

The intestinal epithelium creates a physical and molecular barrier that protects the host from potentially damaging elements in the constantly changing outside environment. Epithelial barrier function is supported by a diverse population of underlying immune cells, which deploy a variety of host defense mechanisms against harmful agents (1). Coordinated signals resulting from microbial sensing, cell-to-cell contact, cytokines, and other chemical mediators determine the type and extent of responses of gut immune cells, balancing tissue homeostasis with effective antimicrobial function via inflammation.

Advances in understanding intestinal physiology, pathophysiology, and host immunity have traditionally relied on studies conducted in animal models (or animal tissue) and in traditional tissue culture systems using colon cancer cell lines. Animal models, including mutant mouse strains, have contributed to the mechanistic understanding of the composition, function, regulatory processes, and operatives of immunity at the gut mucosa. Unfortunately, host restrictions limit the utility and value of animal models (2, 3). This is the case for many enteric pathogens for which small animals fail to recreate disease as it occurs in humans. Likewise, immortalized (transformed) cell lines (e.g., HT-29, Caco-2, and T84) do not reflect human physiological responses but rather the aberrant behavior of diseased cells (e.g., karyotype defects). These cell line-based cultures also lack the multicellular complexity of the human intestinal epithelium, which further reduces the reliability and significance of the data generated.

The establishment of human enteroids from Lgr5^+^ intestinal stem cells was a breakthrough in tissue culture technology (4). Since then, three-dimensional (3D) intestinal enteroids have been widely used as models to study human gut physiology and pathophysiology as well as host-microbe interactions (5–7). Not only do enteroids render a truer physiological representation of the human epithelium, but they also offer a practical and reliable system to probe mechanisms and interventions at the gut mucosal interface. The 3D spheroid conformation can be adapted to produce a 2D monolayer configuration with enteroids seeded on a semipermeable membrane (i.e., Transwell insert) (8–10). An important practical advantage to this simplified format is that it allows direct and controlled access to the apical (mimicking the lumen) and basolateral sides of the epithelial cells, thus facilitating experimental manipulation and evaluation of outcomes. Enteroid monolayers, which can be generated from any gut segment, reflect the undifferentiated (crypt-like) and differentiated (villus-like) intestinal epithelial cell composition (i.e., absorptive enterocytes, goblet cells, enteroendocrine cells, and Paneth cells) (7, 11–14) of the human gut and exhibit segment-specific phenotypic (15) and functional attributes of the normal gastrointestinal epithelium, including production and secretion of mucus (9, 16).

To better recreate the cellular complexity of the gastrointestinal mucosal barrier, we devised a human primary cell coculture system consisting of enteroid monolayers and macrophages seeded on the basolateral side (11). Studies using this enteroid-macrophage coculture model demonstrated physical and cytokine/chemokine-mediated interactions between intestinal epithelial cells and macrophages in the presence of pathogenic Escherichia coli (11, 17). Aiming to expand this coculture conformation to include other phagocytic cells, we established an *ex vivo* coculture model containing intestinal epithelial cells and human primary polymorphonuclear neutrophils (PMN) facing the monolayers’ basal membrane. The histological and functional features of this coculture model, such as cell integration, PMN phenotype, PMN-epithelial cell physical and molecular interactions, and cell function, were characterized. Coordinated epithelial and PMN antimicrobial responses were examined using *Shigella* as a model enteric pathogen. *Shigella* causes diarrhea and dysentery in humans by traversing the colonic barrier via M cells and infecting epithelial cells, and this process involves massive recruitment of PMN (18). The human enteroid-PMN coculture revealed dynamic changes in PMN phenotype triggered by epithelial cell contact in the intestinal environment and modeled a paradoxical role of PMN contributing to inflammation and controlling infection.

## RESULTS

### Establishment of a PMN-enteroid coculture and PMN-epithelial cell interaction.

**FIG 1:**
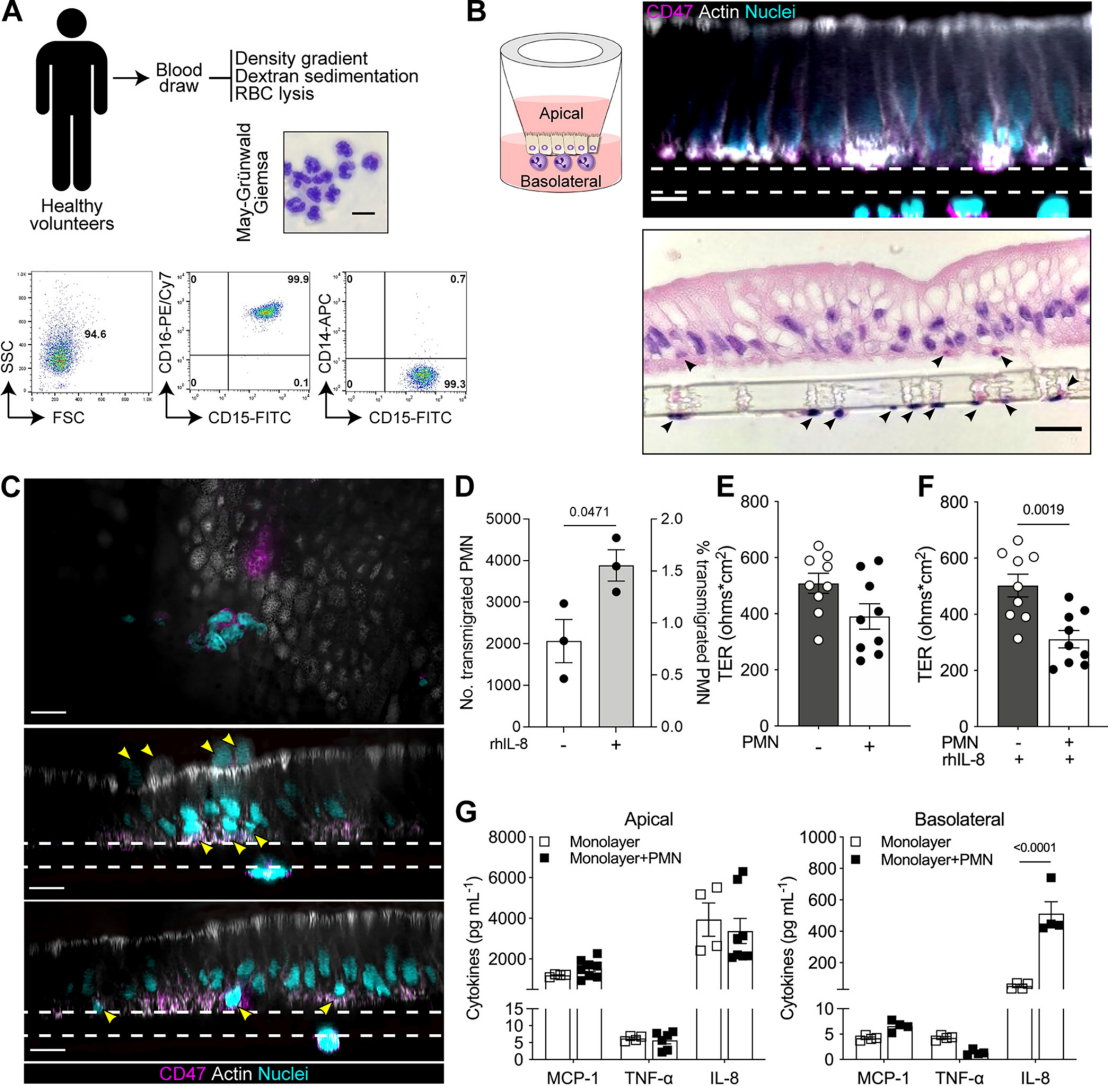
Establishment of a human PMN-enteroid coculture. (A) May-Grünwald-Giemsa-stained image of PMN isolated from peripheral blood (top; bar = 10 μm). Representative scatter plots of PMN phenotype (bottom). (B) Schematic representation of the PMN-enteroid coculture model. Confocal *xz* projection (top; dashed lines, Transwell insert; bar = 10 μm) and H&E (bottom; bar = 20 μm) images of the PMN-enteroid coculture. Arrowheads indicate PMN seeded on the Transwell insert and at the base of the columnar epithelium. (C) Representative immunofluorescence confocal microscopy images (top, *xy* projection from the apical epithelial membrane; middle and bottom, *xz* projections) of enteroid monolayers with PMN intercalated and moving up within the epithelial cell monolayer within 30 min of coculture. Arrowheads indicate PMN. Dashed line, Transwell insert. Bar = 10 μm. (D) Number and proportion of PMN that transmigrated to the luminal compartment 2 h after being added to the coculture in the absence or presence of apically delivered rhIL-8. Percent transmigrated PMN was calculated as follows: number of PMN retrieved in the apical medium/number of PMN attached to the Transwell insert (~2.75 × 10^5^ cells) × 100. Each dot represents the average for three replicate wells; data are shown as mean ± SEM from three independent experiments. (E) TER of enteroid monolayers (gray bar) and PMN-enteroid cocultures (white bar) in a 2 h coculture. (F) TER of enteroid monolayers and PMN-enteroid coculture apically treated with rhIL-8 for 2 h. (E and F) Each dot represents an independent monolayer; data are shown as mean ± SEM from three independent experiments. (G) Cytokines secreted into the apical and basolateral compartments after 2 h of coculture. Data are shown as mean ± SEM from three independent experiments carried out in triplicate. *P* values were calculated by Student’s *t* test.

To interrogate PMN adaptation and function in the human gut, we established an enteroid-PMN coculture model that contains human primary enteroid monolayers and PMN isolated from peripheral blood. A human coculture containing enteroid monolayers and macrophages with similar configuration has been developed by our group (11). Human ileal 3D organoids derived from Lgr5^+^-cell-containing biopsy specimens from healthy subjects were seeded upon the inner (upper) surface of a Transwell insert and allowed to grow until they reached confluence. PMN isolated from peripheral blood of healthy human adult volunteers and exhibiting a CD15^+^ CD16^+^ CD14^−^ phenotype (19) (Fig. 1A) were seeded on the outer (bottom) surface of the insert (Fig. 1B). Confocal immunofluorescence microscopy and hematoxylin-and-eosin (H&E) staining confirmed the coculture’s expected epithelial cell polarity, with the brush border oriented toward the luminal side (apical compartment) and adherent PMN facing the basolateral side of the monolayer (Fig. 1B). Interestingly, the basolaterally seeded PMN rapidly mobilized toward the epithelium. Within 30 min, PMN migrated through the insert’s pores and were intercalated within the epithelial cells (Fig. 1C). The migrating PMN could be retrieved from the apical compartment medium; approximately 1% of the seeded PMN transmigrated across the epithelium (Fig. 1D). The addition of PMN to the enteroid monolayer modestly increased epithelial permeability (a 21% reduction in transepithelial electrical resistance [TER] was observed), although the difference did not reach statistical significance (Fig. 1E). Because interleukin 8 (IL-8) promotes PMN recruitment (20), we examined the effect of exogenous IL-8 on the mobilization of PMN cocultured with epithelial cells and on monolayer permeability. Apical treatment of monolayers with 100 ng/mL of IL-8 significantly increased PMN epithelial transmigration (1.8-fold; 1.5-2% of seeded PMN) (Fig. 1D) and barrier permeability, with a 38% decrease in TER as observed in cocultures containing both PMN and IL-8 (Fig. 1F). Importantly, IL-8 alone did not affect the permeability of monolayers (Fig. 1F).

Since cell movement is influenced by molecular mediators, we next examined the presence of cytokines (pro- and anti-inflammatory) and chemoattractant molecules in tissue culture medium collected from the apical and basolateral compartments of enteroid monolayers alone and of enteroid-PMN cocultures. Basolateral levels of IL-8 produced by the PMN-containing enteroids were 10-fold higher than those of IL-8 produced by the monolayers alone, while monocyte chemoattractant protein (MCP-1) and tumor necrosis factor alpha (TNF-α) remained unaffected by addition of PMN (Fig. 1G). Similarly, the presence of PMN did not affect apical secretion of MCP-1, TNF-α, and IL-8 (Fig. 1G). Production of MCP-1 was distinctly polarized, with higher levels being released to the apical side of the epithelial barrier. IL-1β, IL-6, IL-10, IL-12p70, gamma interferon (IFN-γ), and transforming growth factor β1 (TGF-β1) were measured but determined to be below the limit of detection of the assay for each cytokine.

Taken together, these results demonstrate adequate engraftment of PMN on the basolateral side of the enteroid monolayer, rapid migration of PMN across the monolayer to the luminal side, PMN-induced basolateral secretion of IL-8, and membrane destabilization (increased permeability) by IL-8-enhanced PMN transepithelial movement.

### The human intestinal epithelium environment determines PMN immune phenotype and functional capacity.

**FIG 2:**
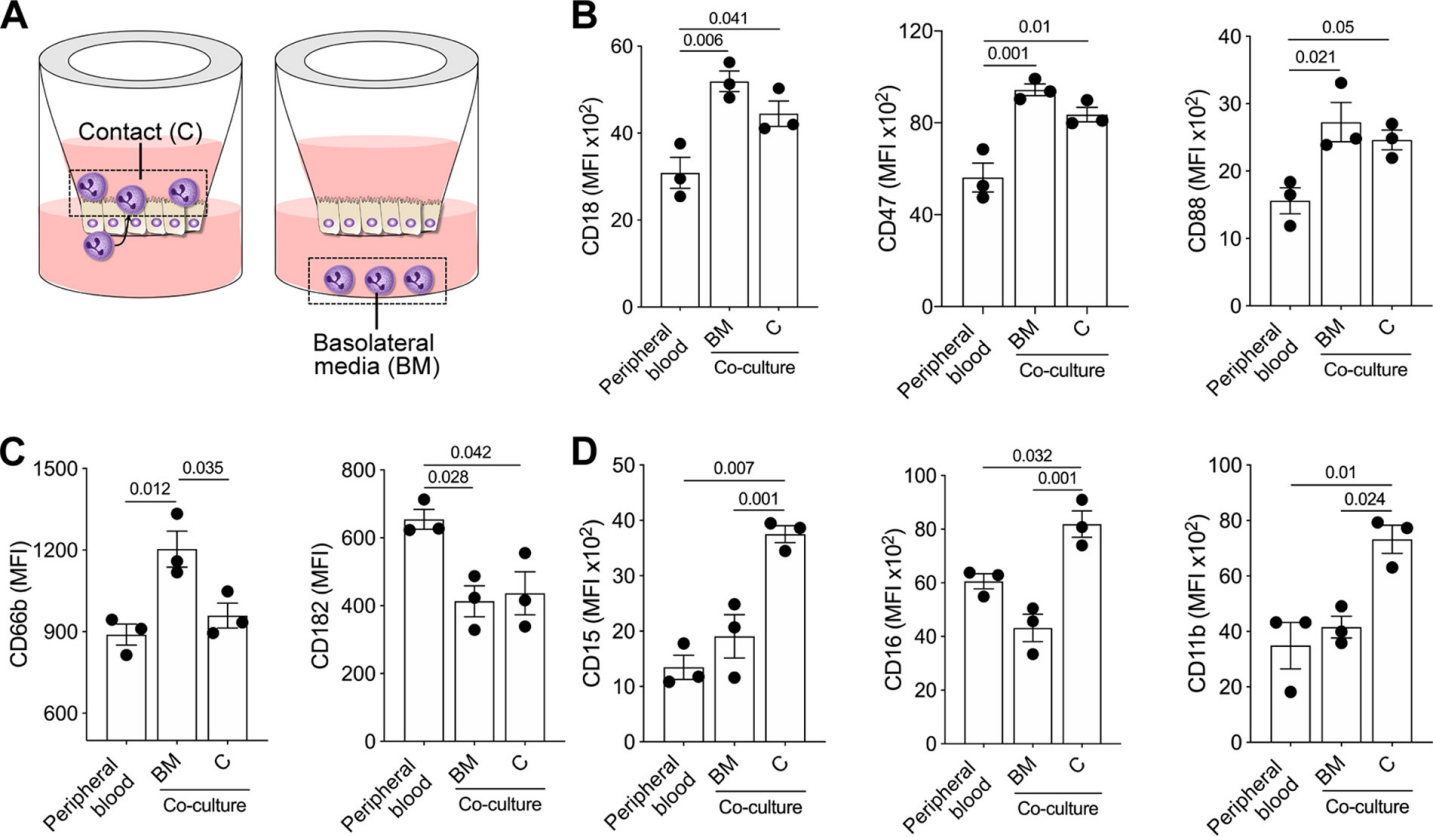
Distinctive phenotype of PMN within the human intestinal environment. (A) Schematic representation of PMN in direct contact with the apical membrane of epithelial cells (C) or in the basolateral medium (BM). (B to D) Phenotypes of isolated PMN, PMN in C, and PMN in the BM determined by flow cytometry within 2 h of coculture. Each dot represents data for three replicate wells; data are shown as mean ± SEM from three independent experiments. *P* values were calculated by one-way ANOVA with Tukey’s post-test for multiple comparisons. The gating strategy and viability plot of PMN cocultured with epithelial cells are shown in Fig. S1A.

Cell phenotype, morphology, and function can be affected by the surrounding tissue and molecular environment. We hypothesized that the immune phenotype and functional capacity of PMN added to the enteroid monolayer would be influenced by their proximity or direct contact with the intestinal epithelium. To explore this hypothesis, we determined the expression of cell surface markers and phenotypic features of PMN isolated from peripheral blood in comparison with those of PMN within the enteroid coculture. Two populations of PMN cocultured with enteroids were investigated: (i) PMN that had been in direct contact with the epithelial cells and traversed the monolayers and (ii) PMN harvested from the basolateral medium as a tissue-adjacent milieu (Fig. 2A). PMN cocultured with enteroid monolayers had a distinct phenotypic profile compared with PMN freshly isolated from peripheral blood. Regardless of their location, whether in contact with cells or in basolateral media, PMN cocultured with enteroid monolayers exhibited increased expression of CD18 (β2 integrin), a molecule that participates in extravasation of circulating PMN, as well as upregulation of CD47, a receptor for membrane integrins involved in cell adhesion and migration, and of CD88 (C5a receptor), a molecule that mediates chemotaxis, granule enzyme release, and superanion production (Fig. 2B; Fig. S1B). CD66b (CEA cell adhesion molecule 8), a marker of secondary granule exocytosis and increased production of reactive oxygen species, was likewise increased, but only in PMN harvested from the basolateral medium (not in contact with cells) (Fig. 2C; Fig. S1B). In contrast, the expression of CD182 (CXCR2 or IL-8RB) was reduced in all PMN in the coculture, regardless of which site they were retrieved from (Fig. 2C; Fig. S1B). PMN that were in close contact with epithelial cells exhibited increased expression of CD15 (E-selectin), a molecule that mediates PMN extravasation; CD16 (FcγRIII), a receptor for IgG that mediates degranulation, phagocytosis, and oxidative burst; and CD11b (α integrin), a protein that facilitates PMN adhesion and, along with CD18, forms the Mac-1 complex implicated in multiple antimicrobial functions (e.g., phagocytosis, cell-mediated cytotoxicity, and cellular activation) (Fig. 2D; Fig. S1B). These results demonstrate that PMN within the intestinal epithelial environment undergo unique phenotypic adaptations, some of which are driven by molecular signals while others require direct PMN-epithelial cell contact.

10.1128/mbio.00944-22.1 FIG S1 Distinctive phenotype of PMN within the human intestinal environment. (A) Representative flow cytometry plots showing PMN viability, and the gating strategy used to determine cell surface expression of CD15, CD16, CD11b, CD18, CD14, CD47, CD54, CD66b, CD88, and CD182 on PMN. (B) Representative histograms showing cell surface markers’ expression on PMN isolated from peripheral blood, PMN that had been in direct contact with the enteroid monolayer (C), or PMN that detached and were retrieved in the basolateral medium (BM). Data are representative of three independent experiments. Download FIG S1, PDF file, 1.0 MB. Copyright © 2022 Lemme-Dumit et al. 2022 Lemme-Dumit et al. https://creativecommons.org/licenses/by/4.0/ This content is distributed under the terms of the Creative Commons Attribution 4.0 International license.

### PMN interaction with *Shigella* as a model enteric pathogen.

**FIG 3:**
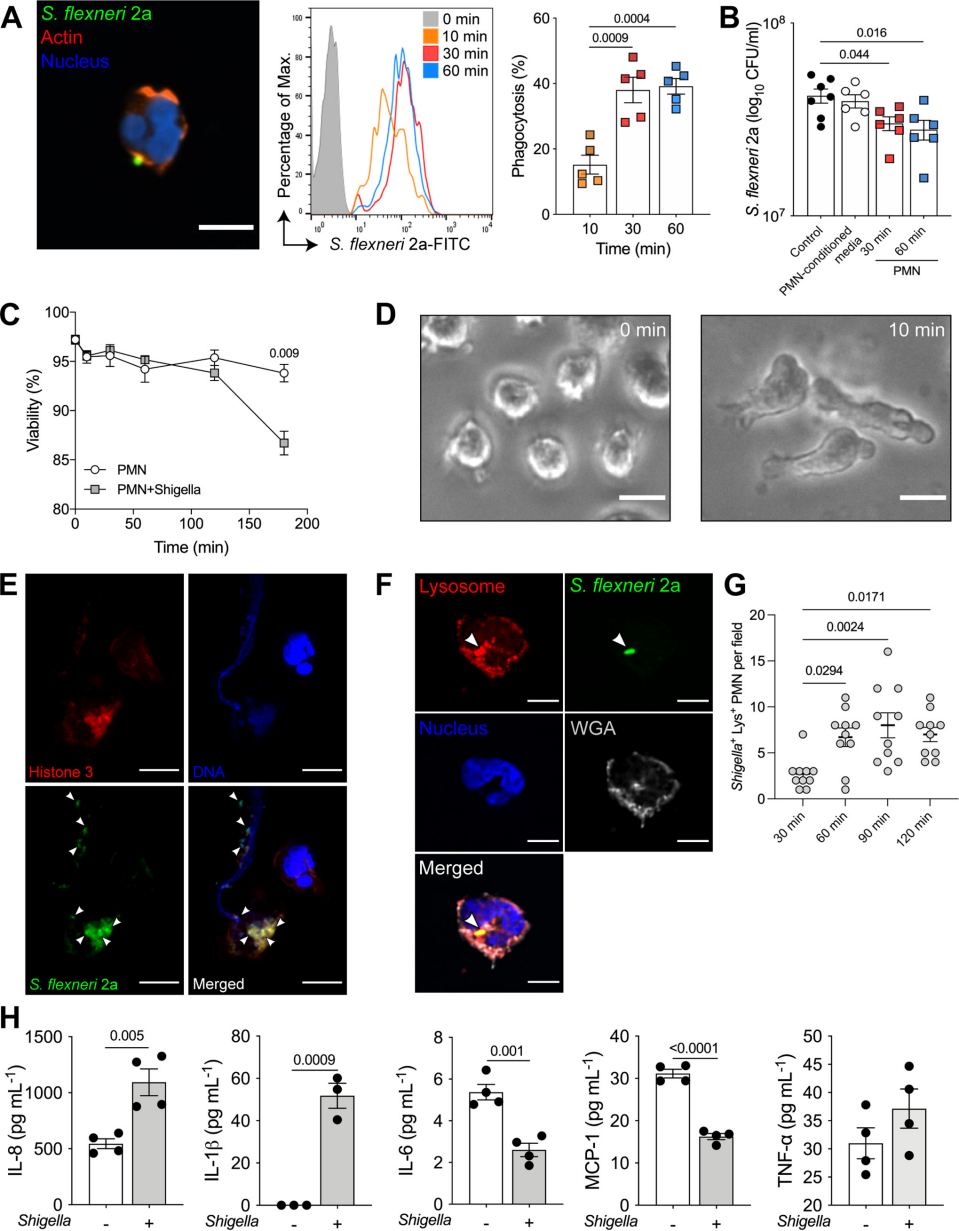
Innate immune responses of human PMN to S. flexneri. (A) Representative confocal microscopy image (left, *xy* projection; bar = 10 μm), histogram of S. flexneri 2a-FITC uptake by human PMN (middle), and percentage of phagocytosis at 10, 30, and 60 min postinfection (left). Each dot represents the average for three replicates and PMN from five individual donors; data are shown as mean ± SEM from three independent experiments. (B) Extracellular S. flexneri 2a CFU in culture medium following PMN exposure to S. flexneri 2a (1 × 10^8^ CFU/mL) for 30 and 60 min; controls included S. flexneri 2a in culture medium or in PMN-conditioned medium without PMN. Each dot represents the average for three replicates and PMN from six individual donors; data are shown as mean ± SEM from three independent experiments. (A and B) *P* values were calculated by one-way ANOVA with Tukey’s post-test for multiple comparisons. (C) PMN viability in the presence and absence of S. flexneri 2a. Data are averages for three replicates and PMN from three individual donors; data are shown as mean ± SEM from three independent experiments. (D) PMN morphology before (0 min) and after (10 min) exposure to S. flexneri 2a. Bars = 10 μm. (E) Confocal microscopy image of NETs 30 min after PMN exposure to S. flexneri 2a. Arrowheads indicate bacteria. Bars = 5 μm. (F) Immunofluorescence image of S. flexneri 2a-FITC colocalization with PMN lysosome 30 min postinfection. Arrowheads indicate bacteria located intracellularly and within the lysosome compartment. Bars = 5 μm. (G) Colocalization of S. flexneri 2a-FITC with PMN 30 min to 2 h postinfection. Each dot represents the number of *Shigella*-positive Lys^+^ PMN per 10 consecutive microscopy fields. *P* values were calculated by one-way ANOVA with Tukey’s post-test for multiple comparisons. (H) Cytokines secreted in culture supernatants of PMN alone and PMN exposed to S. flexneri 2a for 2 h. Data are shown as mean ± SEM from three independent experiments done in triplicate. (C and G) *P* values were calculated by Student’s *t* test.

To interrogate human epithelial cell and PMN interactions in the context of an enteric infection, we exposed the coculture to wild-type (WT) Shigella flexneri 2a (strain 2457T) as a model pathogen. PMN participate in *Shigella* pathogenesis through secretion of proinflammatory cytokines and deploy antimicrobial functions, including phagocytosis, proteolytic enzymes, antimicrobial proteins, and neutrophil extracellular trap (NET) production. We first determined baseline responses of peripheral blood PMN in the presence of *Shigella*. PMN phagocytosed fluorescein isothiocyanate (FITC)-labeled S. flexneri 2a 2457T within 10 min of exposure (Fig. 3A, left); bacterial phagocytosis increased over time (up to 1 h tested), reaching a maximum effect at 30 min (Fig. 3A, middle and right). In parallel, the number of bacteria recovered from the culture supernatant of *Shigella*-exposed PMN decreased significantly within 30- and 60-min exposures, in comparison to the number of bacteria recovered from control wells containing *Shigella* alone (in the absence of PMN) resuspended in tissue culture medium or medium that had been exposed to PMN to control for any soluble bactericidal source (Fig. 3B).

Overall PMN viability was not affected during the first 2 h of *Shigella* exposure but decreased significantly by 3 h postinfection (Fig. 3C; Fig. S2). *Shigella*-exposed PMN exhibited changes in cell morphology and motility; formation of pseudopodia (projections of the cell membrane that enable locomotion) was observed within 10 min postinfection (Fig. 3D). In the presence of *Shigella*, PMN also displayed dynamic amoeboid motility toward the bacteria and released NETs, as evidenced by overlap of DNA and histone 3, with entrapped bacilli (Fig. 3E). FITC-stained *Shigella* organisms colocalized with PMN phagolysosome (Fig. 3F), and the number of *Shigella*-positive Lys^+^ PMN increased over time and remained steady up to 2 h postinfection (Fig. 3G). The observation of NET formation at early stages of infection, likely involved a small number of PMN, as the cell population remained largely viable. In addition, the increase in phagocytic activity later during infection is consistent with functional heterogeneity and plasticity of PMN. It has been hypothesized that stepwise deployment of antimicrobial functions by PMN with intrinsically different phenotypes might be beneficial to fine-tune antibacterial responses in the presence of an acute infection to prevent collateral damage due to hyperactivation (21).

10.1128/mbio.00944-22.2 FIG S2 PMN viability following *Shigella* infection. Representative flow cytometry plot depicting PMN viability 30 and 180 min after *Shigella* infection; uninfected PMN incubated in the same conditions served as controls. Data are representative of three independent experiments. Download FIG S2, PDF file, 0.3 MB. Copyright © 2022 Lemme-Dumit et al. 2022 Lemme-Dumit et al. https://creativecommons.org/licenses/by/4.0/ This content is distributed under the terms of the Creative Commons Attribution 4.0 International license.

In addition, PMN upregulated production and secretion of IL-8 and IL-1β, which are key molecular mediators of *Shigella* pathogenesis, 2 h postinfection, while production of IL-6 and MCP-1 was downregulated; production of TNF-α was not affected (Fig. 3H). IL-10, TGF-β1, and IFN-γ were also measured but were below the limit of detection of the assay. These results showed that PMN antimicrobial responses against *Shigella* involved morphological changes, phagocytic activity, and modulation of inflammatory cytokines.

### Coordinated epithelial cell and PMN responses to *Shigella* infection in the coculture model.

**FIG 4:**
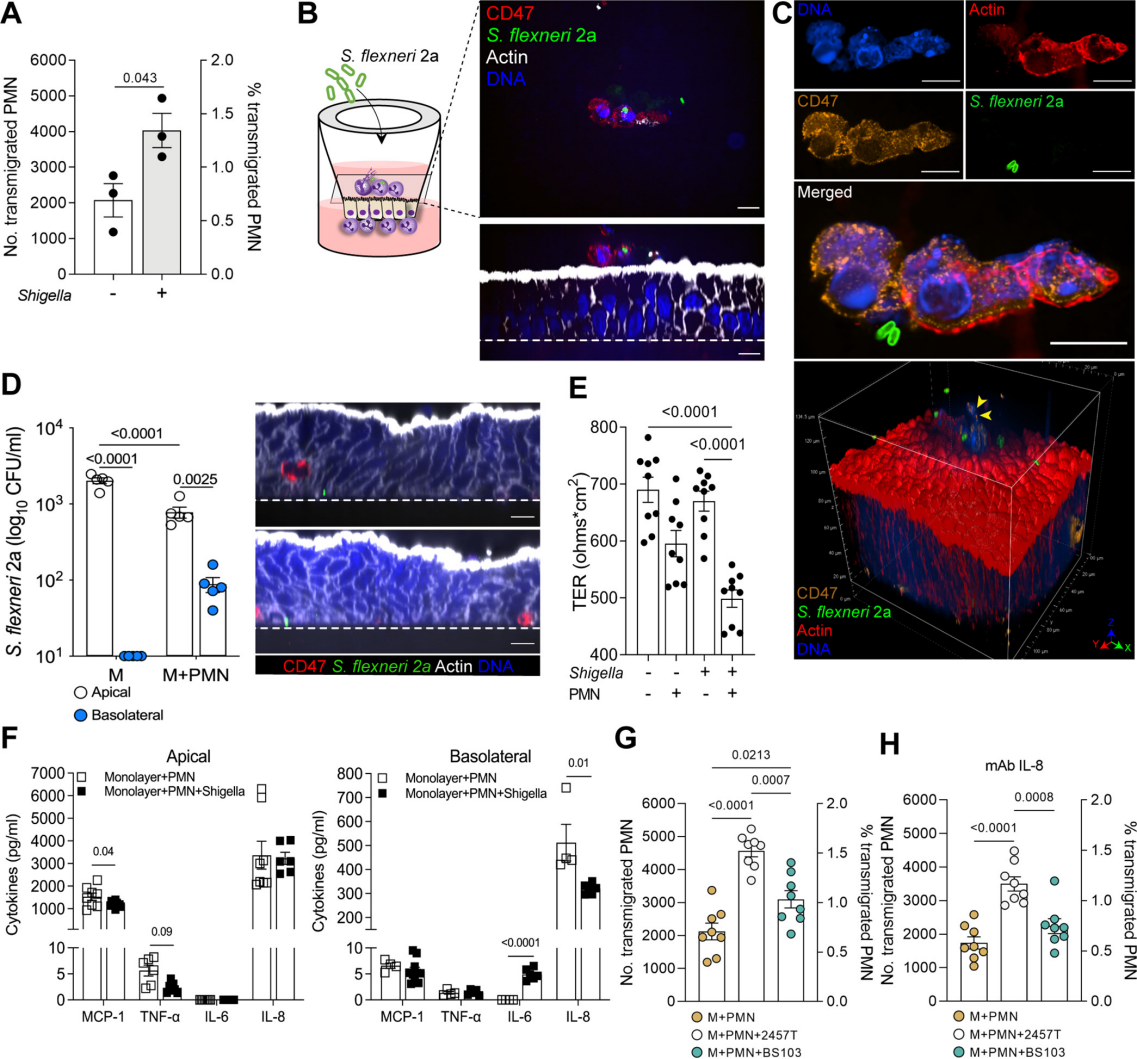
Coordinated innate immune response to *Shigella* by human intestinal epithelial cells and PMN. (A) Numbers and proportion of PMN that transmigrated to the apical compartment of enteroid monolayers exposed or not to S. flexneri 2a WT for 2 h. Percent transmigrated PMN was calculated as follows: number of PMN retrieved in the apical medium/number of PMN attached to the Transwell insert (~2.75 × 10^5^ cells) × 100. (B) Schematic representation (left) and confocal microscopy images (right; *xy* [top] and *xz* [bottom] projections) of PMN-enteroid coculture infected with S. flexneri 2a for 2 h. Dashed line, Transwell insert. Bars = 10 μm. (C) Confocal microscopy images and 3D projection of NET on the apical membrane of PMN-enteroid coculture exposed to S. flexneri 2a for 2 h. Arrowheads indicate decondensed extracellular thread-like DNA. Bars = 10 μm. (D) *Shigella* 2a CFU in the apical and basolateral media of enteroid monolayers (M) and PMN-enteroid cocultures (M+PMN) infected apically for 2 h. Confocal microscopy images (*xz* projections) of PMN-enteroid coculture infected with S. flexneri 2a for 2 h. Dashed line, Transwell insert. Bars = 10 μm. (A and D) Each dot represents the mean for three replicate wells; data are shown as mean ± SEM from three (A) and five (D) independent experiments. *P* values were calculated by Student’s *t* test (A), Student’s *t* test multiple comparisons (D). (E) TER of enteroid and PMN-enteroid cocultures exposed to S. flexneri 2a for 2 h; enteroid monolayer alone or cocultured with PMN for 2 h were included as controls. Each dot represents an independent monolayer; data are shown as mean ± SEM from three independent experiments. *P* values were calculated by one-way ANOVA with Tukey’s post-test for multiple comparisons. (F) Cytokines secreted in the apical and basolateral media of PMN-enteroid cocultures exposed or not to S. flexneri 2a for 2 h. Data are shown as mean ± SEM from three independent experiments in triplicate. *P* values were calculated by Student’s *t* test. (G) Numbers and proportions of PMN that transmigrated to the apical compartment of enteroid monolayers exposed or not to S. flexneri 2a 2457T or BS103 for 2 h. (H) Numbers and proportions of PMN that transmigrated to the apical compartment of enteroid monolayers pretreated with human anti-IL-8 monoclonal antibody and exposed or not to S. flexneri 2a 2457T or BS103 for 2 h. Each dot represents an independent monolayer; data are shown as mean ± SEM from two independent experiments. *P* values were calculated by one-way ANOVA with Tukey’s post-test for multiple comparisons.

We interrogated PMN and epithelial cell interactions and coordinated responses to *Shigella* in the PMN-enteroid cocultures. PMN were added to the enteroid monolayer as described above (Fig. 1B), allowed to settle for 2 h, and then apically exposed to WT S. flexneri 2a (multiplicity of infection [MOI] = 10) for another 2 h. Nonexposed cocultures served as controls. Consistent with our previous observation, PMN facing the basolateral side of the epithelial cells moved swiftly through the filter pores, traversed between the epithelial cells and across the monolayer, and protruded on the apical side. PMN basolateral-apical transmigration (both total number and the proportion) increased in the presence of *Shigella* (Fig. 4A). Confocal immunofluorescent images revealed PMN phagocytosis of bacteria (Fig. 4B; CD47^+^ PMN stained in red, engulfed S. flexneri 2a in green, actin in white, and nuclei in blue) and NET formation with trapped organisms (Fig. 4C; Movie S1) on the luminal surface.

10.1128/mbio.00944-22.5 MOVIE S1 NET on the apical cell membrane of PMN-enteroid coculture exposed to *Shigella*. Three-dimensional projection of the colocalization of decondensed extracellular thread-like DNA (blue), CD47 (orange), and actin (red) with trapped S. flexneri 2a (green). Download Movie S1, MOV file, 15.1 MB. Copyright © 2022 Lemme-Dumit et al. 2022 Lemme-Dumit et al. https://creativecommons.org/licenses/by/4.0/ This content is distributed under the terms of the Creative Commons Attribution 4.0 International license.

The addition of PMN to the enteroid monolayers enabled *Shigella* penetration and cell invasion through the basolateral side (Fig. 4D), whereas in the absence of PMN, *Shigella* was unable to trespass the intact enteroid; no bacteria could be recovered from apically exposed enterocytes (Fig. 4D). TER values were significantly reduced in the *Shigella*-exposed PMN-enteroid coculture compared with *Shigella*-exposed monolayers without PMN or monolayers control (no PMN, no *Shigella*) (Fig. 4E). This observation is consistent with tissue damage and loss of barrier function that enables bacterial translocation. Similar to the results shown in Fig. 1E, TER values in monolayers containing PMN were somewhat lower than that in monolayers alone, although the difference did not reach statistical significance.

Cytokines produced by the *Shigella*-infected and noninfected PMN-enteroid cocultures were also examined in the culture medium collected from the apical and basolateral compartments. *Shigella* infection reduced apical production of MCP-1 and TNF-α by the cocultured cells, increased production of IL-6, and substantially reduced secretion of IL-8 basolaterally (Fig. 4F). IL-6 was only detected in *Shigella*-exposed PMN-enteroid cocultures and secreted exclusively to the basolateral side (Fig. 4F). IL-1β, a hallmark of *Shigella* pathogenesis, was measured but found to be below detectable levels in both apical and basolateral compartments.

Next, we examined whether PMN migration across the epithelial monolayer was dependent on bacterial virulence factors. To this end, PMN-enteroid cocultures were exposed to S. flexneri 2a WT strain 2457T (used in previous experiments as described above) or S. flexneri 2a BS103, avirulent plasmid-cured strain (22). The number and proportion of PMN retrieved from the apical compartment increased significantly in cocultures infected with either strain compared with noninfected controls (Fig. 4G; Fig. S3A). However, PMN transmigration was higher in response to the virulent than the noninvasive strain (Fig. 4G).

10.1128/mbio.00944-22.3 FIG S3 Coordinated response to *Shigella* by human intestinal epithelial cells and PMN. (A) Confocal microscopy images (top, *xy* projections; bottom, *xz* projections) of PMN-enteroid cocultures apically exposed to S. flexneri 2a 2457T (left) or BS103 (right) for 2 h. Bar = 20 μm. (B) Total amount of IL-8 in the apical and basolateral media of PMN-enteroid cocultures pretreated or not with anti-IL-8 monoclonal antibody and exposed or not to S. flexneri 2a 2457T or BS103 for 2 h. Data are shown as mean ± SEM from two independent experiments done in duplicate. Download FIG S3, PDF file, 0.9 MB. Copyright © 2022 Lemme-Dumit et al. 2022 Lemme-Dumit et al. https://creativecommons.org/licenses/by/4.0/ This content is distributed under the terms of the Creative Commons Attribution 4.0 International license.

To evaluate whether PMN transmigration upon infection was influenced by a molecular (cytokine) gradient, PMN-enteroid cocultures were pretreated with anti-IL-8 monoclonal antibody and infected with S. flexneri 2a 2457T or BS103 as described above. Blocking of IL-8 in the apical and basolateral culture media was confirmed by enzyme-linked immunosorbent assay (ELISA) (Fig. S3B). PMN basolateral-to-apical transmigration still increased in cultures infected with WT *Shigella* under IL-8 blocking conditions (although the proportion of transmigrated PMN was lower) but not in those exposed to the plasmid-cured *Shigella* BS103 strain (Fig. 4H).

Collectively, these observations demonstrate that *Shigella* infection causes active recruitment of PMN from the basolateral side of the epithelium (where they were seeded) to the luminal side, and this process involves bacterial virulence factors and host molecular mediators such as epithelial cell-derived IL-8. Paradoxically, the PMN activation and transmigration produced epithelial barrier damage that enabled *Shigella* penetration and basolateral infection; meanwhile, PMN actively engulfed bacteria and increased production and secretion of inflammatory cytokines to the apical and basolateral compartments.

### Phenotypic changes in PMN cocultured with epithelial cells in response to *Shigella* infection.

**FIG 5:**
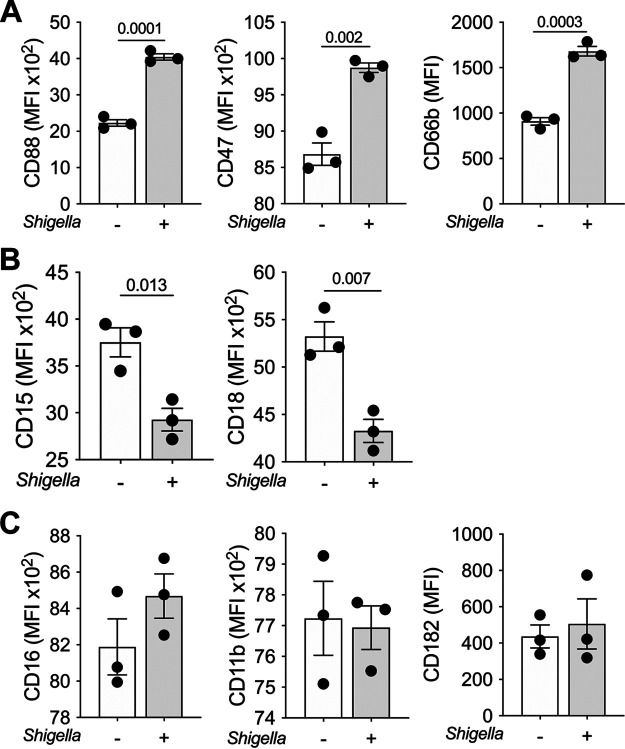
Immune phenotype of PMN cocultured with epithelial cells and exposed to *Shigella*. (A to C) Cell surface expression of CD15, CD16, CD11b, CD18, CD88, CD66b, CD47, and CD182 on PMN embedded in the enteroid monolayer (coculture) exposed or not to S. flexneri 2a for 2 h. Each dot represents data from three replicate wells; data are shown as mean ± SEM from three independent experiments. *P* values were calculated by Student’s *t* test. The gating strategy and viability plot of PMN cocultured with epithelial cells and exposed to S. flexneri 2a WT are provided in Fig. S4.

Additional experiments were conducted to determine the phenotypic features of PMN cocultured with epithelial cells upon *Shigella* infection. PMN cocultured with intestinal epithelial cells and exposed to *Shigella* exhibited increased expression of CD88, CD47, and CD66b compared with PMN in cocultures that remained uninfected (Fig. 5A; Fig. S4B). In contrast, CD15 and CD18 expression was decreased on PMN from *Shigella*-infected cocultures (Fig. 5B; Fig. S4B). CD16, CD11b, and CD182 remained unchanged (Fig. 5C; Fig. S4B). These results suggest that PMN residing within the intestinal environment undergo immune phenotypic adaptation as a result of pathogen exposure consistent with increased antimicrobial function (i.e., activation and expression of molecules that facilitate chemotaxis and epithelial transmigration).

10.1128/mbio.00944-22.4 FIG S4 Immune phenotype of PMN cocultured with epithelial cell monolayers and exposed to *Shigella*. (A) Representative flow-cytometric PMN live/dead plot and (B) contour plots and histograms depicting cell surface markers’ expression on PMN cocultured with enteroid monolayers and exposed (or not) to S. flexneri 2a for 2 h; PMN were retrieved from the apical side. Data are representative of three independent experiments. Download FIG S4, PDF file, 0.7 MB. Copyright © 2022 Lemme-Dumit et al. 2022 Lemme-Dumit et al. https://creativecommons.org/licenses/by/4.0/ This content is distributed under the terms of the Creative Commons Attribution 4.0 International license.

## DISCUSSION

Epithelial cells and innate phagocytic cells underlying the intestinal epithelium work synergistically, preventing the trespassing of harmful agents and deploying rapid and effective host defense mechanisms against pathogens. PMN are the first innate immune cells recruited in response to gastrointestinal tissue inflammation and infection, and they play a critical role in initiating host immune responses (23). Patients with neutrophil disorders are prone to recurrent microbial infection (24, 25). In this paper, we report the successful establishment of an *ex vivo* primary human intestinal epithelial cell-PMN coculture, and we describe cell interactions, phenotypic and functional adaptations, and cellular and molecular innate responses to *Shigella*, a relevant dysenteric pathogen.

Our group developed the first *ex vivo* human enteroid and monocyte-derived macrophage coculture model in a monolayer format (11). The same approach was used to produce the PMN-enteroid coculture described herein. Unlike macrophages, which remained in the basolateral side of the monolayer (where seeded) and responded to luminal organisms by extending transepithelial projections between adjacent epithelial cells, PMN rapidly migrated from the basolateral side of the epithelial cell and across the monolayer via the paracellular space. Histological and confocal microscopy images revealed PMN crawling through the Transwell insert pores, embedding at the base of the epithelial cells, and emerging on the luminal side of the enteroid monolayer, all within a few hours of coculture. While macrophages contributed to cell differentiation and stabilized the epithelial barrier in our previous studies, PMN transmigration resulted in increased barrier permeability that enabled bacterial invasion.

The capacity of PMN to migrate across the vascular endothelium (26) and a variety of tissues (27, 28), including epithelial cell layers (29–32), has been documented *in vivo* (mainly in animal models) or *in vitro* using cell lines. These processes have been associated with PMN activation as a result of microbial sensing, inflammation, or danger signals. The level of myeloperoxidase (MPO), one of the principal enzymes contained in PMN granules and released upon activation is, in stool, a biomarker of inflammatory bowel disease severity (33). In our human PMN and intestinal epithelial cell coculture model, PMN migrated even in the absence of external stimulatory signals. PMN are not typically present in the homeostatic gut but actively recruited by signs of inflammation or infection (34); therefore, unprovoked PMN migration would not be expected.

The tissue microenvironment can influence immune cell phenotype and effector function capabilities (35, 36). PMN from peripheral blood exhibited rapid phenotypic changes when incubated with enteroid monolayers. They acquired an activated phenotype that was triggered by tissue-derived signals. Compared to the PMN from peripheral blood, PMN cocultured with epithelial cells had increased expression of CD18, which favors cell binding; of CD88, which is the receptor for C5a and facilitates degranulation and chemotaxis; and of CD47, a cell surface glycoprotein that supports transmigration across endothelial and epithelial cells (37–39). PMN phenotypic changes were influenced by their spatial location, i.e., whether they were in direct contact with epithelial cells or simply present in basolateral culture medium. PMN in close proximity to the epithelium (the migratory PMN) had upregulated expression of CD15, which participates in chemotaxis and extravasation from circulation; as well as FcγRIII (CD16), a low-affinity Fc receptor for IgG; and CD11b, a marker of cell adhesion and antimicrobial function (phagocytosis, degranulation, and oxidative burst) (40, 41). On the other hand, PMN which were retrieved from the basolateral medium, and which had not been in contact with the epithelial layer, exhibited increased expression of CD66b, indicative of PMN activation and degranulation (42). This is, to our knowledge, the first detailed description of dynamic changes of PMN immune phenotype in a translationally relevant model of the human intestinal epithelium.

MCP-1/CCL2, a chemoattractant and enhancer of bacterial killing and survival of phagocytic cells, and IL-8, a hallmark product of intestinal epithelial cells and a potent activator and chemoattractant for PMN (43, 44), were abundantly produced by the ileal monolayers in our coculture model. TNF-α, a recruiter and activator of phagocytic cells (45), was also detected, albeit at lower levels. IL-8 was further sourced by the PMN within the coculture and released to the basolateral medium; *in vivo*, this subepithelial surge of IL-8 likely contributes to PMN recruitment *in vivo*. Intriguingly, expression of IL-8 receptor (CD182/CXCR2) was downregulated in PMN cocultured with enteroid monolayers compared to PMN from peripheral blood, most likely reflecting a compensatory mechanism. Still, IL-8 may act via another high-affinity receptor (i.e., CXCR1 or IL-8RA) (46, 47). Expression of MCP-1, IL-8, and TNF-α has been reported in intestinal tissue of healthy adults in steady state (48, 49).

Again, clear differences in innate immune functions emerged when cytokine profiles in PMN- and macrophage-enteroid cocultures were compared. Macrophages contributed to high levels of IFN-γ and IL-6 (11); however, these cytokines were not detected in cocultures containing PMN. Accordingly, both models were capable of discerning distinct morphological as well as functional phagocytic cell adaptation.

*Shigella* invades the human colon and rectal mucosa and causes severe inflammation, massive recruitment of PMN, and tissue destruction (18). Bloody/mucous diarrhea (dysentery) with large numbers of PMN in stool is a hallmark of shigellosis (50). Human intestinal enteroids can be infected with *Shigella* (51, 52). Hence, our model was fitting to interrogate coordinated innate responses of epithelial cells and PMN to this enteric pathogen. *Shigella* added to the apical side of the enteroid monolayers increased basolateral-to-apical PMN migration. Early efflux of PMN into the colonic tissue has been observed during shigellosis in the infected rabbit loop model (53). Perdomo and colleagues reported *Shigella*-induced PMN transmigration that promoted invasion of colonic T84 cell (54). The same group later showed that the *Shigella* lipopolysaccharide transcytosed to the basal side of T84 cells enhanced adherence of subepithelial PMN through IL-8 signaling (55). In the present study, we demonstrated that IL-8 enhanced PMN transepithelial migration across human intestinal epithelial cells in culture. However, the fact that PMN still migrated under IL-8 blocking conditions indicates that signals other than IL-8 are likewise involved in this process. In a series of articles, McCormick and colleagues interrogated molecules and bacterial components involved in *Shigella*-PMN responses in T84 cells (56–59); the group reported the contribution of hepoxilin A3, a product of the cleavage of arachidonic acid via 12/15-lipoxygenase (56) and the requirement of S. flexneri plasmid-encoded virulence effectors for PMN migration (60–62). In contrast, Perdomo et al. found intermediate PMN migration in T84 monolayers exposed to a noninvasive (plasmid-cured) *Shigella* strain (54). Our results align with the latter and confirm that virulence factors contribute to but are not the only determinants of PMN transepithelial migratory signaling.

During *Shigella* dysentery, PMN migration through the colonic epithelium destabilizes the epithelial barrier and allows massive entry of bacteria into the submucosa with further amplification of infection and tissue destruction (63). This process was recreated in our coculture model; PMN transmigration across the intestinal monolayer disrupted the polarized epithelial barrier and enabled bacterial invasion. Focal breakdown of the epithelial cell surface has been attributed to PMN migration in various disease states, including infectious enterocolitis (64). Intestinal epithelial repair events (e.g., cell proliferation and migration and closure of leaking epithelial lateral spaces) reportedly begin minutes after acute mucosal barrier injury (65). As a corollary of these observations, we are investigating epithelial repair subsequent to PMN-induced inflammation and cell disruption and the mechanisms and elements involved.

Modeling the human PMN-epithelial cell interaction *ex vivo*, our findings challenge the notion that M cells are necessary for *Shigella* epithelial translocation and invasion and hint that the infiltration and barrier disruption by PMN offer an alternative mechanism by which *Shigella* and other invasive enteric pathogens access the host internal compartment. We are presently studying reverse transmigration of bacterium-loaded PMN (out of the lumen and back to the basolateral side) as a possible means to initiate adaptive immunity through cross-presentation. The PMN-enteroid coculture model is also useful for interrogating molecules involved in PMN activity in the context of enteric infections and to identify targets to prevent intestinal injury and inflammation.

Although acting in a “brute force” manner, PMN deployed potent antimicrobial activity against *Shigella*. As expected, circulating PMN trapped bacteria in NET structures and exhibited *Shigella* phagocytic activity. PMN antimicrobial functions coincided with increased production of IL-8 and the pyroptosis inducer IL-1β and downregulation of IL-6 and MCP-1. Likewise, PMN embedded within the epithelial cells promptly mobilized upon sensing *Shigella* on the epithelial cell surface; PMN that had traversed to the apical side formed NET structures that trapped bacteria and exhibited robust phagocytic and killing capacity. Intriguingly, IL-8 levels were reduced, IL-1β was absent, and IL-6 was increased in the infected PMN-epithelial cell cocultures compared to noninfected cocultures. A reduction of IL-8 production had been reported in *Shigella*-infected human colonic explants, which was ascribed to anti-inflammatory bacterial proteins or death of IL-8-secreting cells (66). Reduced levels of these inflammatory cytokines may also reflect negative feedback to prevent further tissue damage. Heightened levels of IL-6 and reduced TNF-α during infection may suggest a protective epithelial mechanism after injury (67, 68). In addition, IL-6 has been ascribed a beneficial role in enhancing Th17 protective immunity against *Shigella* reinfection (69). Because cytokines measured in the coculture supernatant represent the total amount produced by diverse cell types, this readout is limited in its capacity to discern subtle differences between culture conditions.

The immune phenotype of PMN in the coculture adapted again as a result of *Shigella* infection, with further increases in the activation/granule-associated markers CD66b, CD88, and CD47. CD47 has been implicated in PMN paracellular migration through epithelial cells in response to the bacterium-derived leukocyte chemoattractant *N*-formyl-methionyl-leucyl-phenylalanine, in a process that involves intracellular distribution and increased CD47 cell surface expression (38). CD47-deficient mice have increased susceptibility to E. coli as a result of reduced PMN trafficking and bacterial killing activity (70). This finding is consistent with our observed upregulation of CD47 in *Shigella*-exposed PMN, which is likely associated with PMN’s antimicrobial activity. CD16 and CD11b expression were unaltered on PMN cocultured with intestinal enteroids and exposed to *Shigella*, indicating a preserved phagocytic capacity, whereas extravasation and cell adhesion markers CD15 and CD18 were downregulated. It has been reported that CD47 expression increases gradually and modulates CD11b-integrin function and CD11b/CD18 surface expression on PMN, suggesting a regulatory mechanism between these molecules (38, 71). Expression of CD47 is self-protective; it avoids clearance by phagocytic cells (72). The exact role of CD47 expression on PMN during *Shigella* infection remains to be elucidated.

Human intestinal xenografts in immunodeficient mice have been used to model interactions of *Shigella* with the human intestine *in vivo* (73). The model failed to discern any role of PMN in ameliorating or exacerbating disease but revealed larger numbers of intracellular bacteria in PMN-depleted mice; the authors concluded that while PMN may contribute to tissue damage, they are important in controlling bacterial dissemination. The combination of species, immunodeficient background, and impracticality are major confounders/limitations of this model (73).

Our study contributed new insights into the morphological, phenotypic, and functional adaptation of PMN in the gastrointestinal environment, the close communication between PMN and epithelial cells, and their coordinated responses to *Shigella* as a model enteric pathogen. The human PMN-enteroid coculture described here provides a translationally relevant *ex vivo* model to study human epithelial cell-PMN physiology and pathophysiology, as well as host cell interactions and innate responses to enteric organisms. This model could be useful to interrogate innate immune defense mechanisms against enteric pathogens and to support the development and evaluation of preventive or therapeutic tools.

## MATERIALS AND METHODS

### Human PMN isolation.

Human peripheral blood was collected in EDTA tubes (BD Vacutainer) from healthy volunteers enrolled in University of Maryland Institutional Review Board (IRB) approved protocol HP-40025-CVD4000, and methods were conducted in compliance with approved Environmental Health and Safety guidelines (IBC 00003017). PMN were obtained by Ficoll-Paque (Premium solution; GE Healthcare Bio-Sciences AB, Sweden) gradient centrifugation following dextran (Alfa Aesar, USA) sedimentation (74). Contaminating erythrocytes were removed by hypotonic lysis. After being washed, cells were suspended in enteroid differentiation medium (DFM) without antibiotics and immediately used. The cell suspension contained >95% PMN, as determined by flow cytometry and May-Grünwald-Giemsa-stained cytopreparations. PMN viability was >98%. Cell counts were determined using the Guava ViaCount reagent (Luminex, USA); viable and nonviable cells were distinguished based on differential permeabilities to two DNA-binding dyes. Cells were stained following the manufacturer’s instructions and analyzed in Guava 8HT using ViaCount software (Luminex, USA).

### Preparation of enteroid monolayers.

Human enteroid cultures were established from biopsy tissue obtained after endoscopic or surgical resection from healthy subjects at Johns Hopkins University under Johns Hopkins University IRB approved protocol NA-00038329, as previously described (14). Briefly, enteroids generated from isolated intestinal crypts from ileal segments were maintained as 3D cysts embedded in Matrigel (Corning, USA) in 24-well plates and cultured in Wnt3A-containing nondifferentiated media (NDM) (74). Multiple enteroids were harvested with Culturex organoid harvesting solution (Trevigen, USA), and small enteroid fragments were obtained to create 2D monolayers by digestion with TrypLE Express (Thermo Fisher) in a 37°C water bath for 90 s. Enteroid fragments were resuspended in NDM containing 10 μM Y-27632 and 10 μM CHIR 99021 inhibitors (Tocris) (NDM+inhibitors). The inner surfaces of Transwell inserts (3.0-μm-pore transparent polyester membrane) precoated with 100 μL of human collagen IV solution (34 μg/mL; Sigma-Aldrich, USA) were seeded with 100 μL of an enteroid fragment suspension, and 600 μL of NDM+inhibitors was added to the wells of a 24-well tissue culture plate and incubated at 37°C and 5% CO_2,_ as previously described (74). NDM without inhibitors was replaced after 48 h, and fresh NDM was added every other day; under these conditions, enteroid cultures reached confluence in 14 to 16 days. Monolayer differentiation was induced by incubation in Wnt3A-free and Rspo-1-free DFM without antibiotics for 5 days (11). Monolayer confluence was monitored by measuring TER values with an epithelial voltohmmeter (EVOM^2^; World Precision Instruments, USA). The unit area resistances (ohms·cm^2^) were calculated according to the growth surface area of the inserts (0.33 cm^2^).

### PMN-enteroid coculture.

Differentiated enteroid monolayers seeded on Transwell inserts were inverted and placed in an empty 12-well plate. PMN (5 × 10^5^ in 50 μL of DFM) were added onto the bottom surface of the inserts, and cells were allowed to attach for 2 h at 37°C and 5% CO_2_ (inserts remained wet throughout this process). The inserts were then turned back to their original position into a 24-well plate, and DFM was added to the insert (100 μL) and to the well (600 μL). Approximately 45% of the added PMN remained attached to the Transwell insert. TER measurements were collected after 2 h, allowing monolayer recovery. For the IL-8 neutralization experiments, 0.4 μg/mL of human IL-8 monoclonal antibody (R&D Systems, USA) was added to the apical and basolateral compartments of enteroid monolayers and to the PMN suspensions before they were cocultured. This dose of anti-IL-8 was approximately 100-fold above the highest value of IL-8 detected in the tissue culture media of *Shigella*-exposed PMN-enteroid cocultures. The absence of IL-8 in enteroid and PMN culture media was confirmed by electrochemiluminescence ELISA, as described in the cytokine measurement section below.

### Shigella flexneri 2a strains and infection.

Shigella flexneri 2a WT strain 2457T and avirulent plasmid-cured strain BS103 were grown from frozen stocks (−80°C) on tryptic soy agar (TSA) (Difco BD, USA) supplemented with 0.01% Congo red dye (Sigma-Aldrich) overnight at 37°C. A bacterial inoculum was made by resuspending single red (2457T) and white (BS103) colonies in sterile 1× phosphate-buffered saline (PBS) (pH 7.4) (Quality Biological). Bacterial suspension was adjusted to the desired concentration (~1 × 10^8^ CFU/mL) in advanced Dulbecco’s modified Eagle medium F-12 (DMEM/F-12) without serum. A bacterial suspension containing ~5 × 10^6^ CFU in 50 μL was added directly to PMN (for 30 to 60 min) or to the apical compartment of enteroid monolayers alone or PMN-enteroid coculture (for 2 h), at a multiplicity of infection of 10 relative to 1 PMN.

### PMN transmigration and apical and basolateral harvesting.

Basolateral-to-apical PMN transmigration was quantified by measurement of PMN MPO using a commercial kit (Cayman Chemical, Ann Arbor, MI) as previously described (75). The assay was standardized with a known number of human PMN. MPO activity in lysates of enteroid monolayers alone was negligible. Transmigrated PMN were also confirmed by counting cells on the apical membrane of enteroid monolayers of 25 consecutive immunofluorescence microscopy fields. For IL-8 gradient experiments, 100 ng/mL of recombinant human IL-8 (rhIL-8) (BioLegend, San Diego, CA) were added to the apical side of the enteroid monolayers (76), and PMN transmigration was determined as described above.

PMN that migrated across the monolayer were collected from the apical media by washing the apical compartment three times with 1× PBS at room temperature (RT). PMN that were not in contact with the epithelial cells were harvested from the basolateral tissue culture media.

### PMN phagocytosis.

S. flexneri 2a WT cultures grown overnight as described above were washed, resuspended in sterile PBS, and incubated with FITC (Sigma-Aldrich) (20 μg/mL) for 30 min at 37^0^C. The bacterial suspension was thoroughly washed and adjusted to ~10^8^ CFU/mL in sterile PBS-glycerol (1:2) and stored at −80°C until used. The day of the assay, FITC-labeled *Shigella* was incubated with PMN-autologous human sera for 30 min at 37°C. Opsonized bacteria (5 × 10^6^ CFU) were added to PMN suspensions (5 × 10^5^ cells) and incubated for 10, 30, and 60 min. Phagocytosis was measured by flow cytometry. External fluorescence was blocked with the addition of trypan blue, and the difference between the mean fluorescence intensities (MFI) of blocked and nonblocked samples was used to calculate percent of phagocytosis (77, 78).

### H&E and immunofluorescence staining.

PMN-enteroid coculture cells were fixed in aqueous 4% paraformaldehyde (PFA; Electron Microscopy Sciences, USA) at RT for 45 min and then washed with PBS. For H&E staining, monolayers were kept for at least 48 h in formaldehyde solution, then embedded in paraffin, sectioned, mounted on slides, and stained with H&E. For immunofluorescence, cells were permeabilized and blocked for 30 min at RT in PBS containing 15% fetal bovine serum (FBS), 2% bovine serum albumin (BSA), and 0.1% saponin (all from Sigma-Aldrich, USA). Cells were rinsed with PBS and incubated overnight at 4°C with primary antibodies: mouse anti-CD16 (LSBio, USA) and rabbit anti-S. flexneri 2a (Abcam, USA) diluted 1:100 in PBS containing 15% FBS and 2% BSA. Stained cells were washed with PBS and incubated with secondary antibodies: goat anti-mouse AF488 and goat anti-rabbit AF594 (both from Thermo Fisher Scientific, USA) diluted 1:100 in PBS for 1 h at RT; phalloidin AF594 or AF633 (Molecular Probes, Thermo Fisher Scientific) was included in this step for actin visualization. Cells were washed and mounted in ProLong Gold antifade reagent with DAPI (Cell Signaling Technology, USA) for nuclear staining and maintained at 4°C. Lysosome was stained with LysoTracker red DND-99 (Thermo Fisher Scientific) following the manufacturer’s instructions.

### Immunofluorescence microscopy.

Confocal imaging was carried out at the Confocal Microscopy Facility of the University of Maryland School of Medicine using a Nikon W1 spinning disk confocal microscope running NIS-Elements imaging software (Nikon). Images were captured with a 40× or 60× oil objective, and settings were adjusted to optimize signal. Immunofluorescence imaging (Fig. 3F) was carried out using EVOS FL imaging systems (fluorescence microscope) with a 40× lens objective. Images were collated using FIJI/ImageJ (NIH). Signal processing was applied equally across the entire image. Color channels for Fig. 1B and C and for actin (Fig. 4B) were arranged for contrast purposes.

### Flow cytometry.

PMN phenotype was determined using the following human specific monoclonal antibodies: from BD Pharmingen, HI98 (anti-CD15, FITC conjugated), M5E2 (anti-CD14, allophycocyanin [APC] conjugated), 3G8 (anti-CD16, phycoerythrin [PE]/Cy7 conjugated), and D53-1473 (anti-CD88, BV421 conjugated), and from BioLegend, TS1/18 (anti-CD18, PE/Cy7 conjugated), ICRF44 (anti-CD11b, BV421 conjugated), 5E8/CXCR2 (anti-CD182, APC conjugated), CC2C6 (anti-CD47, PE/Cy7 conjugated), HA58 (anti-CD54, APC conjugated), and G10F5 (anti-CD66b, Pacific Blue conjugated). All staining was performed in the dark. For all experiments, PMN were stained with 2 μL of Zombie Aqua dye (BioLegend) diluted 1:100 for 15 min at RT. PMN were washed and blocked with 2% normal mouse serum (Thermo Fisher Scientific) for 15 min at 4°C. After being washed, cells were resuspended in FACS buffer (PBS containing 0.5% BSA and 2 mM EDTA; all from Sigma-Aldrich), and 100 μL of equal numbers of cells was dispensed in several tubes and stained with antibodies for 30 min at 4°C. Antibodies were used diluted 1:2 to 1:1,000; optimal amount was determined by in-house titration. Cells were washed in FACS buffer and either analyzed or fixed in 4% PFA for 15 min at 4°C and analyzed the next day. Marker expression was analyzed in a Guava 8HT using Guava ExpressPro software (Luminex, USA) or BD LSRII using FACSDiva software (BD Biosciences, USA) and analyzed with FlowJo software (v10; Tree Star).

### Cytokine and chemokine measurements.

Cytokines and chemokines were measured by electrochemiluminescence microarray using commercial assays (Meso Scale Diagnostic, USA) following the manufacturer’s instructions. IFN-γ, IL-1β, IL-6, IL-10, IL-12p70, TNF-α, MCP-1, TGF-β1, and IL-8 concentrations in the apical and basolateral culture supernatants were reported in picograms per milliliter.

### Statistical analysis.

Statistical significances were calculated using unpaired Student’s *t* test and one-way or two-way analysis of variance (ANOVA) with Tukey’s post-test as appropriate. Plots and statistical tests were performed using Prism software v9 (GraphPad, San Diego, CA). Treatment comparisons included at least three replicates and three independent experiments. Differences were considered statistically significant at *P* values of ≤0.05. Exact *P* values are indicated for each figure. Results are expressed as mean standard errors of the mean ( ±SEM).
