# Supplementary figures and images for: Epithelial and Neutrophil Interactions and Coordinated Response to Shigella in a Human Intestinal Enteroid-Neutrophil Coculture Model
Source: mBio. 2022 Jun 2;13(3):e00944-22. doi: 10.1128/mbio.00944-22 (PMC9239269; doi:10.1128/mbio.00944-22)

# Supplementary Figure 1

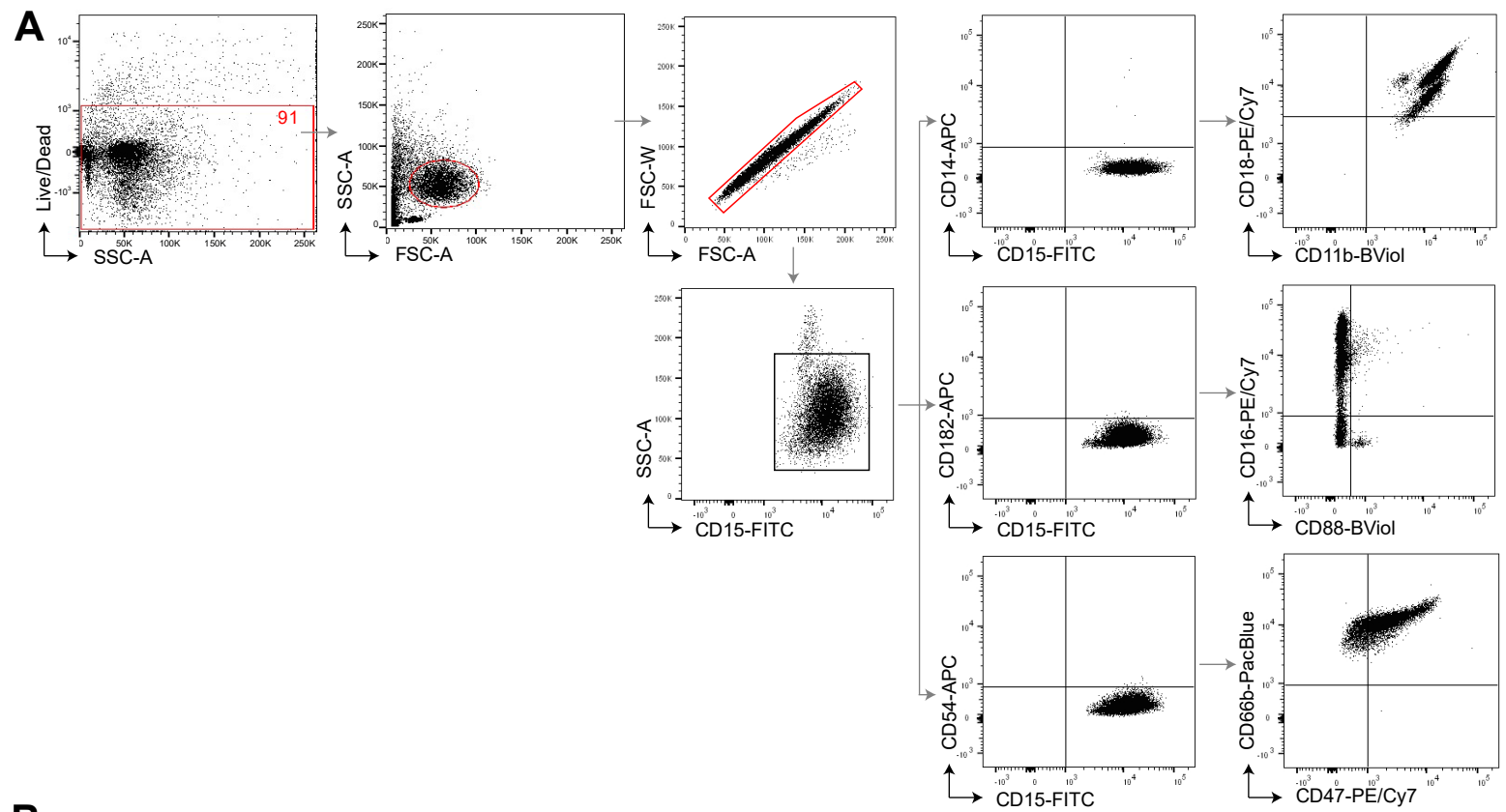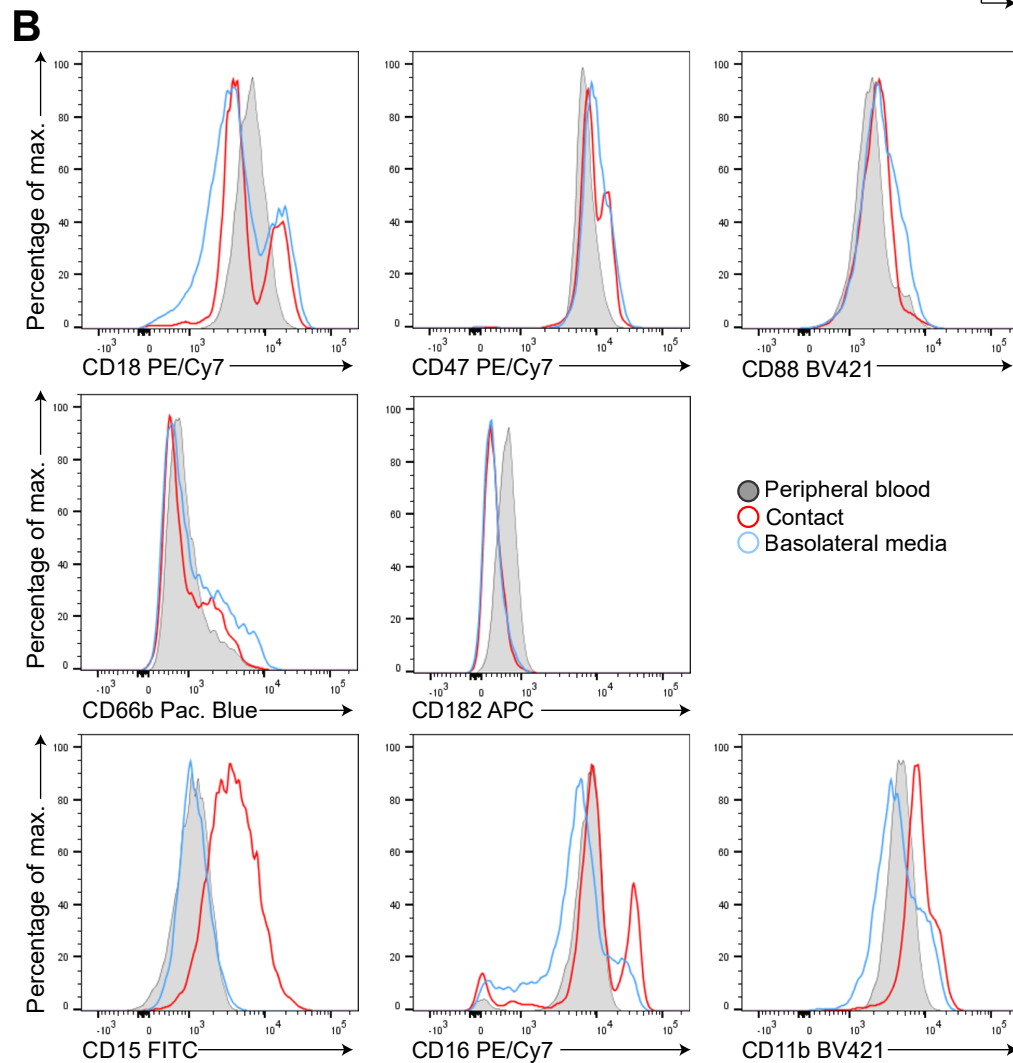

Supplement: FIG S1 [file mbio.00944-22-s0001.pdf]

# Supplementary Figure 2

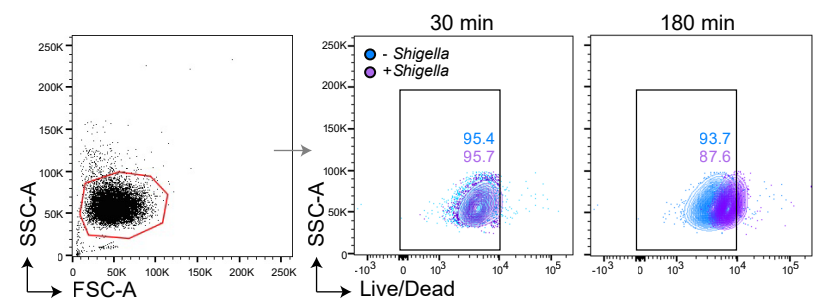

Supplement: FIG S2 [file mbio.00944-22-s0002.pdf]

# Supplementary Figure 3

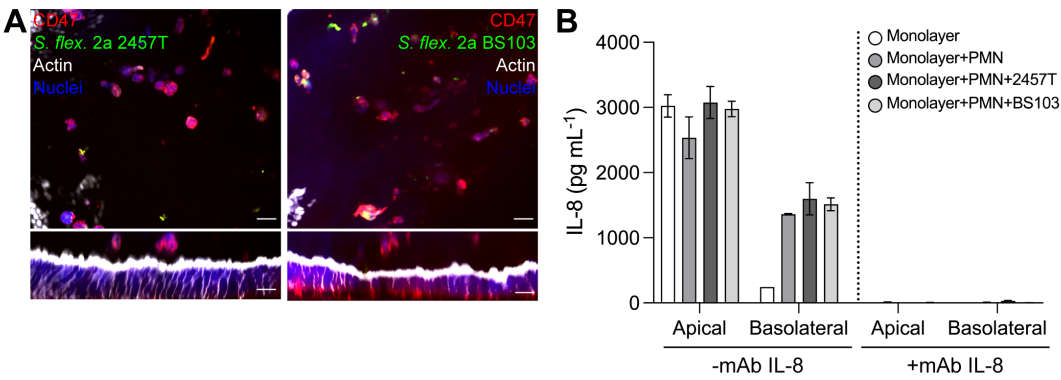

Supplement: FIG S3 [file mbio.00944-22-s0003.pdf]

## Supplementary Figure 4

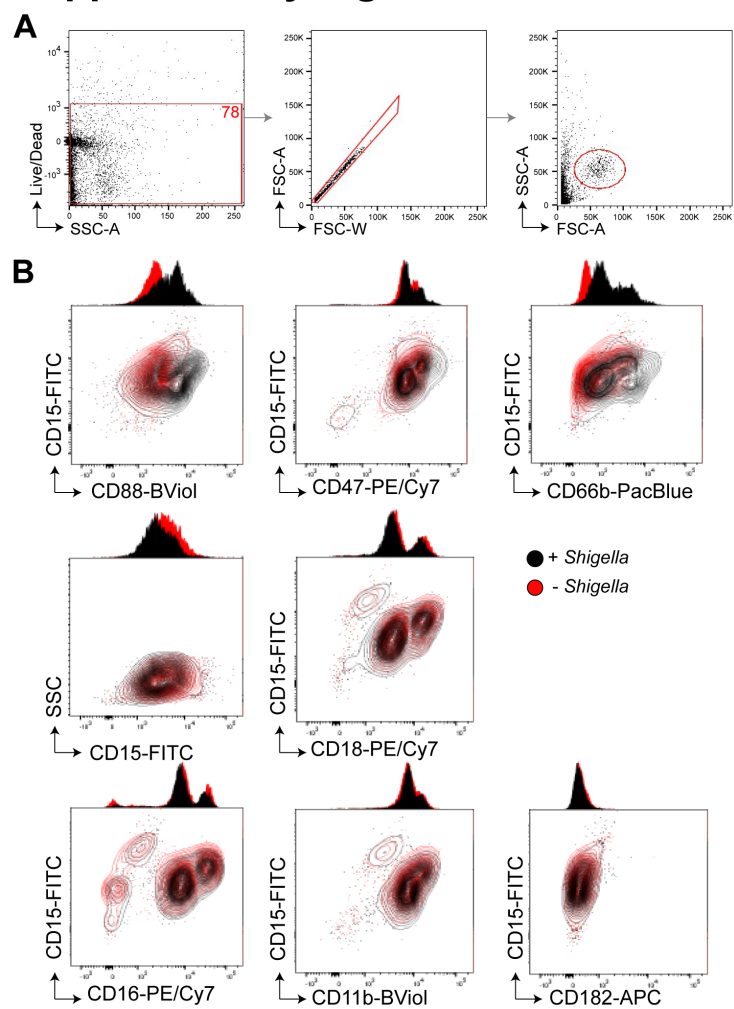

Supplement: FIG S4 [file mbio.00944-22-s0004.pdf]
